# The Use of Ivy

**Published:** 1886-01

**Authors:** 


The Uses of Ivy.—*Land and Water* says the common belief that ivy trained against the walls of a dwelling-house produces damp walls and general unhealthiness, is fallacious. The very opposite is the case. If one will carefully examine an ivy-clad wall after a shower of rain, he will notice that while the overlapping leaves have conducted the water from point to point until it has reached the ground, the wall beneath is perfectly dry and dusty. More than this, the thirsty shoots, which force their way into every crevice of the structure which will afford a firm hold, act like suckers, in drawing out any particles of moisture for their own nourishment. The ivy, in fact, acts like a great coat, keeping the house from wet and warm. One more virtue it has, in giving to the ugliest structure an evergreen beauty.

